# Fluoride exposure and metabolic alterations: a scoping review of metabolomic studies

**DOI:** 10.1007/s11306-025-02353-w

**Published:** 2025-10-10

**Authors:** Guillermo Tamayo-Cabeza, Gina Castiblanco-Rubio, E. Angeles Martínez-Mier

**Affiliations:** Department of Dental Public Health and Dental Informatics, Indiana University School of Dentistry, 1140 W Michigan St, Indianapolis, IN 46202 USA

**Keywords:** Fluoride, Metabolomics, Metabolite, Pathway, Review

## Abstract

**Background:**

Evidence from in-vitro and animal studies suggests that fluoride exposure may alter key metabolic pathways such as amino acid, fatty acid and energy metabolism in different tissues, requiring an understanding of its impact at the molecular level, especially in human populations.

**Aim of review:**

This scoping review aims to systematically map and synthesize the available evidence on metabolic alterations associated with fluoride exposure, specifically focusing on studies employing metabolomic analysis techniques to identify altered metabolites and metabolic pathways at the cellular, tissue, and organ levels.

**Key scientific concepts of review:**

Fluoride exposure has been found to alter a broad range of metabolic pathways, including those involved in energy metabolism (glycolysis, TCA cycle, mitochondrial activity), macromolecule metabolism (purine and fatty acid metabolism, amino acid pathways), and cellular stress responses (oxidative stress and glutathione metabolism). However, there is limited evidence in humans and potential mechanistic studies. While supportive, the reliance on animal models and in-vitro studies points to the need for human studies to compare metabolic alterations at different levels of fluoride exposure to aid in understanding the systemic effects of fluoride on human health.

**Supplementary Information:**

The online version contains supplementary material available at 10.1007/s11306-025-02353-w.

## Introduction

Fluoride is a naturally occurring and widely available anion of the element fluorine, ubiquitously present as a trace element in the human diet (Zohoori & Buzalaf, 2022). Due to its role in preventing and controlling dental caries, it has been added to public water supplies, table salt, milk, dietary supplements, and dental products. However, excess fluoride ingestion can lead to dental and skeletal fluorosis (DenBesten & Li, 2011; Veneri et al., 2023). In addition to its effects on hard tissues, associations between fluoride exposure and adverse effects on cognitive function and behavior have also been reported (Bashash et al., 2017; Green et al., 2019).

Fluoride exposure has been shown to induce metabolic alterations at the cellular, tissue, and organ levels. For example, in mouse bone marrow stem cells, it has been linked to disturbances in amino acid metabolism, which plays a critical role in cellular functions such as proliferation, differentiation, and self-renewal (Wang et al., 2022). Fluoride exposure leads to metabolic changes in serum, kidney, liver, brain, and heart tissues, disrupting amino acid and fatty acid metabolism, as well as energy metabolism pathways (Zhao et al., 2022). Additionally, fluoride exposure has been associated with alterations in the intestinal microbiota (Zhou et al., 2023). However, the translation of these results from in-vitro and animal studies to humans remains limited, suggesting the need for further research and exploration of the potential fluoride-induced metabolomic alterations in humans. Metabolomic approaches, as part of *omics* strategies, offer a high-throughput, comprehensive analysis of metabolites and their interactions, facilitating the identification of subtle changes in metabolic pathways and biomarkers. These approaches are particularly suited for studying fluoride-induced alterations, as they unveil molecular mechanisms and systemic impacts, providing insights into the associations between fluoride exposure and metabolic disruptions.

This scoping review aimed to map and synthesize evidence on metabolic alterations linked to fluoride exposure, focusing on studies using metabolomic analysis to identify altered metabolites and pathways at cellular, tissue, and organ levels.

## Methods

The scoping review was conducted following the frameworks by Arksey and O’Malley (2005), and Levac et al. (2010).

### Protocol and registration

Our protocol was prepared using the Preferred Reporting Items for Systematic Reviews and Meta-analysis (PRISMA) extension for scoping reviews, which was revised by the research team. The final protocol was registered prospectively with the Open Science Framework on May 5, 2023 (10.17605/OSF.IO/928EH).

### Eligibility criteria

Included studies measured metabolites in any sample from human, animal, or cellular models, and their association with fluoride exposure from any source. Specifically, studies investigating metabolic changes associated with fluoride exposure using metabolomic analysis techniques, such as mass spectrometry or nuclear magnetic resonance, with a focus on altered metabolites and metabolic pathways. Peer-reviewed studies were included if they: (1) were primary research studies, such as experimental and observational, that used metabolomic analysis techniques to investigate the impact of fluoride exposure on metabolism; and (2) included the assessment of fluoride exposure from various sources; In vivo or in vitro studies were included with no filter criteria based on years of publication. Only studies available in English, or with English translations, were included.

### Information sources

To identify potentially relevant studies, the following bibliographic databases were searched from May 2023 to February 2024: PubMed/Medline, Embase, Scopus and Web of Science. In addition to published studies, conference abstracts, theses, dissertations, and reports were also searched using Open Grey. The final search strategy for each database can be found in Supplementary material. The search results for each database were exported into EndNote^®^ to remove duplicates.

### Search strategy, selection of sources of evidence, and data charting process

The search terms used were: (fluoride* OR fluorine) AND (metabolomic* OR metabonomic* OR metabolome* OR metabolic profile*).

Search results were imported to Covidence^®^ software for managing and streamlining systematic reviews (Covidence). Using this software, publications were screened, titles and abstracts evaluated, and the full text of all publications was identified.

A data-charting form was developed to identify key data. Data from eligible studies were charted in Covidence ^®^ using a standardized data extraction tool designed for this study.

### Data items

The data extraction tool captured the relevant information on the study’s citation: authors, title, journal, and year of publication; study design and methodology: study population, sample size, and aim of the study; fluoride exposure: source, dose, duration, assessment method and description of control group; metabolomic technique, metabolites or metabolic pathways displaying changes, study limitations, key findings, and conclusions.

### Synthesis of results

We grouped the studies by the study population: human subjects research, studies using animal models, and studies utilizing in-vitro models (Tables 1, 2 and 3). The studies were summarized by design, sample size, fluoride exposure, description of control group(s), fluoride assessment method, metabolomic technique implemented, and key findings. Because descriptions of the fluoride assessment method were only found in human subject research studies, this was omitted for animal and in-vitro studies.

**Table 1:** Human subject research studies included

Author/s (year)	Study characteristics	Fluoride exposure	Fluoride assessment method	Metabolomic technique	Altered metabolites	Altered metabolic pathways
Usman et al. (2022)	Case-control study, including 39 patients with dental and skeletal fluorosis from Tharparkar, Sindh, Pakistan.	Chronic exposure to fluoride in ground water. The control group included 20 participants without chronic exposure to fluoride in ground water.	Ion-selective electrode (ISE) method for determining the concentration of fluoride in serum.	Untargeted metabolomics using Ultra-Performance Liquid Chromatography - Quadrupole Time-of-Flight Mass Spectrometry/Mass Spectrometry (UPLC- QTOF-MS/MS) analysis.	Inosine, α-linolenic acid, guanosine, octanoyl-L-carnitine, His-Trp, phytosphingosine, lauroyl-L-carnitine, hydrocortisone, deoxyinosine, and dodecanedioic acid.	Energy metabolism, fatty acid oxidation, the purine degradation pathway, protein degradation, and ω−6 fatty acid linoleate signatures.
Wang et al.(2023)	Case-control study, including 32 patients with skeletal fluorosis from Guizhou, China	Chronic exposure to fluoride from coal burning. The control group included 33 individuals without fluorosis nor chronic exposure.	Not specified.	Untargeted metabolomics using High-Performance Liquid Chromatography (HPLC) and Electrospray Ionization-Mass Spectrometry (ESI-MSN).	Gut microbiota-derived tryptophan metabolites, particularly tryptamine, 5-hydroxyindoleacetic acid, and indoleacetaldehyde.	Arginine biosynthesis, protein and vitamin digestion and absorption, ABC transporters, sphingolipid metabolism, and several amino acid metabolism pathways.

**Table 2:** Studies of animal models included

Author/s (year)	Controlled animal experiment characteristics	Fluoride source and duration	Metabolomic technique	Altered metabolites	Altered metabolic pathways
Tian et al. (2023)	Sprague Dawley rats, with a total of 24 serum samples collected.	Sodium fluoride (100 mg/L) in drinking water through their mothers during gestation and lactation, and then until postanal day 120. Controls received distilled water.	Nontargeted serum metabolomics using a HPLC system coupled with a mass spectrometer.	Glutamine, alpha-ketoglutarate, and phosphatidylethanolamine (PE).	Proximal tubule bicarbonate reclamation pathway and the autophagy-animal pathway.
Yue et al. (2020)	Sprague Dawley rats, with total of twenty pups, with ten in the fluoride exposed group and ten in the control group.	Sodium fluoride (100 mg/L) in drinking water and through their mothers during lactation. Controls received distilled water.	Ultra-Performance Liquid Chromatography (UPLC) with Tandem Mass Spectrometry (MS/MS).	Nicotinamide, adenosine, 1-Oleoyl-sn-glycero-3-phosphocholine, and 1-Stearoyl-sn-glycerol 3-phosphocholine, while urea, N2-Acetyl-L-ornithine, and betaine.	Amino acid metabolism (including protein digestion and absorption), the urea cycle, choline metabolism, and glycerophospholipid metabolism.
Ba et al. (2022)	Sprague Dawley rats, including six offspring rats in the dental fluorosis group and eight in the control group	Sodium fluoride (100 mg/L) in drinking water from gestation, lactation and post- weaning (three months). Controls received pure water.	LC-MS/MS and gas chromatography-mass spectrometry (GC-MS)	Nicotinamide, adenosine, 1-oleoyl-sn-glycero-3-phosphocholine, and 1-stearoyl-sn-glycerol 3-phosphocholine in serum.	HIF-1 signaling pathway and the glycolysis/gluconeogenesis pathway were the metabolic pathways most significantly correlated with dental fluorosis.
Wang et al. (2023b)	Ross 308 broilers, with a total of 120 1-day-old, divided into four groups of 30 each.	Dietary fluoride as 500 mg/kg (low group), 1000 mg/kg (medium group), and 2000 mg/kg (high fluoride group) for 42 days. Controls were fed dietary fluoride at 0 mg/kg.	Metabolite extraction and analysis was performed using the online platform of the Majorbio Cloud Platform.	Glutathione and malondialdehyde levels.	Glutathione metabolic pathway and the FOXOs pathway.
Zhang et al. (2023b)	Sprague-Dawley rats. A total of 12 rats were used for biological sample collection, six in the fluoride exposed group and six controls.	Sodium fluoride (100 mg/L) in drinking water from the first day of mating until postnatal day 90. Controls received water without added fluoride.	LC-MS and UPLC.	Compounds within the tryptophan and lipoic acid metabolic pathways, such as xanthurenic acid, 5-hydroxyindoleacetic acid, and (R)-lipoic acid.	Linoleic acid metabolism, tryptophan metabolism, lipoic acid metabolism, and α-linolenic acid metabolism.
Zhao et al. (2022)	Sprague Dawley rats, with a total of 12 rats designated equally into a fluoride exposed group and a control group.	Sodium fluoride (100 mg/L) in drinking water administered for 6 weeks. Controls received water without added fluoride.	GC-MS	Amino acids (such as proline, ornithine, and glutamine), fatty acids (like arachidonic and palmitic acid), and organic acids involved in energy metabolism.	Amino acid metabolism (specifically alanine, aspartate, glutamate, arginine, and proline metabolism), glutathione metabolism, and the citrate (TCA) cycle.
Zhou et al. (2023)	Sprague-Dawley rats, with a total of 19 offspring rats, including 9 in the fluoride exposed group and 10 controls.	Sodium fluoride (100 mg/L) in drinking water from mating until 90 days after birth, while the control group received pure water.	LC-MS/MS	α-ketoglutaric acid and UDP-glucose.	Pentose and glucuronate interconversion pathway.
Zhu et al. (2024)	C57BL/6 J mice, including 12 offsprings in each group of fluoride exposed and control groups.	Sodium fluoride (10 or 50 mg/L) in drinking water, for 30-days pre-gestation, and then during pregnancy, lactation and postnatal day 30. Controls received with distilled water.	LC-MS	Choline-related compounds (such as cytidine 5’-diphosphocholine, phosphatidylcholine, lysophosphatidylcholine, and acetylcholine), arachidonic acid and its metabolic precursors, glutamate, and related neurotransmitters.	Choline and arachidonic acid metabolism pathways.

**Table 3:** Studies of In-vitro models included

Author/s (year)	In vitro experiment characteristics	Fluoride source and duration	Metabolomic technique	Altered metabolites	Altered metabolic pathways
Sakagami et al. (2014)	HSC-2 human oral squamous cell carcinoma cell line, with metabolomic profiling of cells conducted twice in triplicate and mediums once in triplicate.	Sodium fluoride with a dose of 8 mM, for various times up to 3 h. Controls cells were treated without sodium fluoride.	Capillary electrophoresis-time of flight mass spectrometry (CE-TOF-MS)	Glycolytic intermediates such as fructose 1,6-phosphate and 2-phosphoglycerate. AMP/ATP ratio, and markers of oxidative stress such as oxidized glutathione (GSSG) and methionine sulfoxide.	Glycolysis pathway and the tricarboxylic acid (TCA) cycle,
Wang et al. (2022)	C57BL/6J mouse bone marrow mesenchymal stem cells (BMSCs), with three biological replicates for transcriptomics and six for metabolomics analysis.	Sodium fluoride was applied to BMSCs at 1, 2, and 4 mM for 24 h, with untreated cells serving as control.	LC-MS.	Palmitic acid, prostaglandin C2, and prostaglandin B2, as well as L-glutamic acid and pyroglutamic acid.	Glutathione metabolism, D-glutamine and D-glutamate metabolism, and alanine, aspartate and glutamate metabolism.
Zhang et al. (2023a)	Human umbilical vein endothelial cells (HUVECs), with at least three separate experiments performed for quantitative analysis.	Sodium fluoride was administered at 75 µmol/L for 24 h, based on a 50% cell viability threshold, with an untreated control group.	LC-MS/MS	Lumichrome and S-Methyl-5’-thioadenosine. Creatine, L-Glutamate, and various lipids, including Stearic acid and Arachidic acid.	FoxO signaling pathway, apoptosis, and the PI3K/Akt signaling pathway.

## Results

### Selection of sources of evidence

**Fig. 1:**
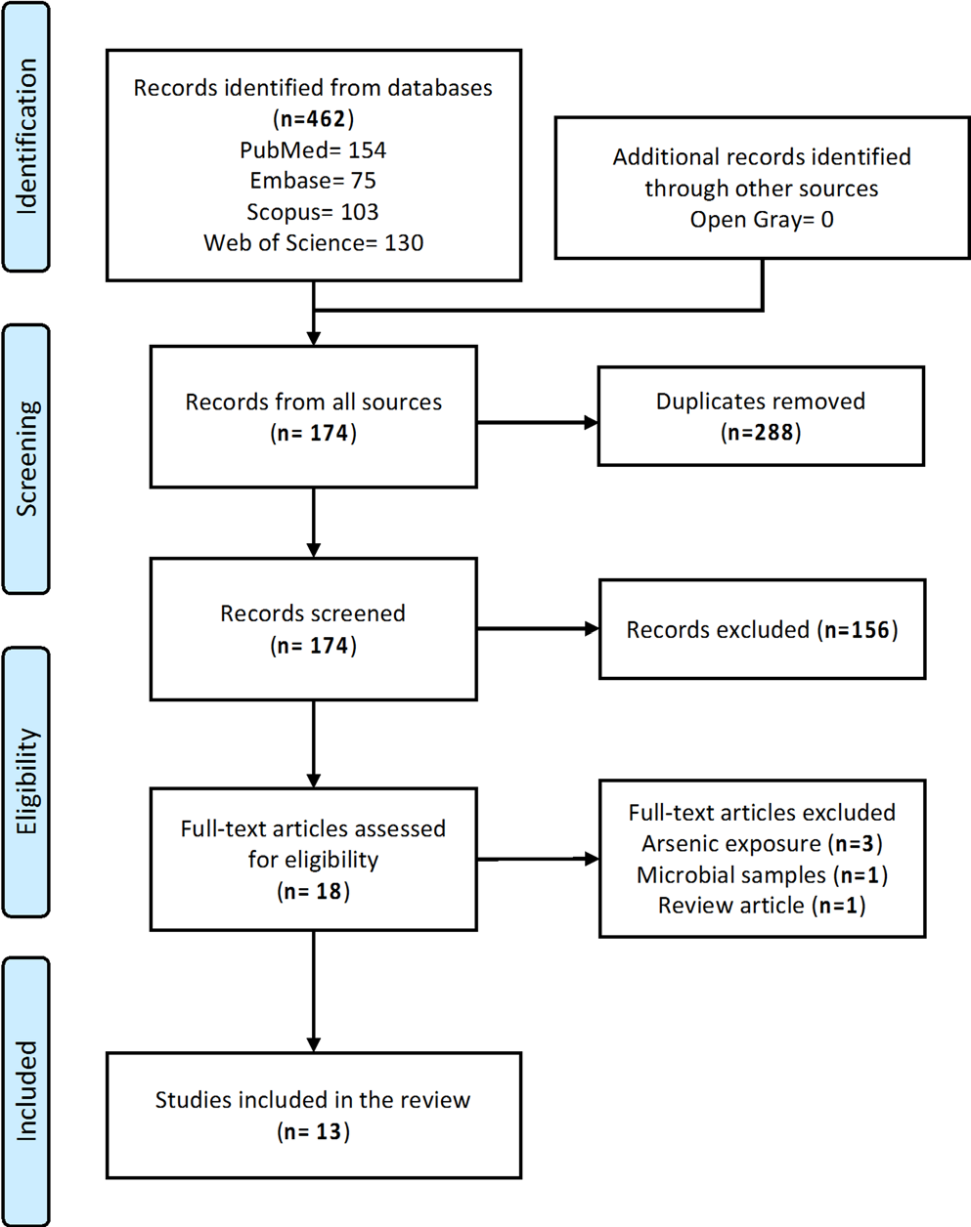
PRISMA flow diagram for study selection

After removal of duplicates, a total of 174 citations were identified from searches of electronic databases (Fig. 1). Based on their titles and/or abstracts, 156 were excluded, with 18 full text articles retrieved and assessed for eligibility. Of these, five articles were excluded for the following reasons: three examined metabolomic changes but in relation to arsenic exposure, one was a clinical trial of human participants examining the effects of stannous fluoride dentifrice in reducing plaque microbial virulence, and another was a narrative review of the literature focusing on pharmaceutical aspects of fluorinated compounds. The remaining 13 studies were considered eligible for this review and had publication years ranging from 2014 to 2024.

### Characteristics of the sources of evidence

Human studies, such as those by Usman et al. (2022) in Pakistan and Wang et al. (2023b) in China, used case-control designs to examine populations affected by dental or skeletal fluorosis due to high fluoride concentrations in groundwater or exposure to coal-burning areas. Only one human study specified using a fluoride assessment method, including the Ion-Selective Electrode (ISE) method (Usman et al., 2022). The metabolomic analyses were performed using liquid chromatography-mass spectrometry (LC-MS), employing untargeted high-resolution tandem mass spectrometry.

Animal studies used controlled animal experimental designs to evaluate fluoride’s effects on metabolic alterations. The animal models primarily consisted of Sprague Dawley rats, with the exception of one study using Ross 308 broilers (Wang et al., 2023a) and another using C57BL/6 J mice (Zhu et al., 2024). A common dose provided in the group of animals experimentally exposed to fluoride was 100 mg/L sodium fluoride in drinking water, with control groups provided either distilled or pure water. The primary metabolomic techniques included LC-MS and gas chromatography-mass spectrometry (GC-MS).

The in-vitro studies examined the cellular effects of fluoride exposure. All studies used sodium fluoride as treatment source. Research on human oral squamous cell carcinoma cells (Sakagami et al., 2014), mouse bone marrow mesenchymal stem cells (Wang et al., 2022), and human umbilical vein endothelial cells (Zhang et al., 2023a), used metabolomic techniques such as capillary electrophoresis-time of flight mass spectrometry (CE-TOF-MS), and LC-MS.

### Individual sources of evidence

Two studies investigated the impact of fluoride exposure on human populations. Usman et al. (2022) conducted a case-control study in Tharparkar, Pakistan, finding metabolic changes in individuals with chronic exposure to high fluoride concentrations in untreated groundwater. The metabolomic alterations found in the case group included disturbances in the purine degradation pathway, decreased levels of corticosteroids, elevated protein degradation, increased omega-6 fatty acid linoleate signatures, and decreased mitochondrial activity. Wang et al. (2023b) focused on skeletal fluorosis in Guizhou, China, finding dysregulated intestinal microflora and altered metabolite levels in individuals exposed to coal-burning areas. These studies suggest a potential link between fluoride exposure and metabolic dysfunctions, with impacts on various pathways and systems, including purine degradation, corticosteroids, protein degradation, intestinal microflora, and carbohydrate metabolism.

Eight controlled animal experiments explored the effects of fluoride exposure on various biological systems. One study revealed that excessive fluoride exposure in Sprague Dawley rats altered 68 metabolites and affected 15 metabolic pathways, showing significant impacts on organ function and metabolism (Zhao et al., 2022). Another study found that fluoride exposure in pregnant rats and their offspring influenced serum metabolites, including amino acid metabolism and specific pathways including protein digestion, amino acid biosynthesis, and urea cycle (Yue et al., 2020). A separate study by the same team (Ba et al., 2022) observed that excessive fluoride intake in Sprague Dawley rats altered serum nicotinamide levels, adenosine, 1-oleoyl-sn-glycero-3-phosphocholine, and 1-stearoyl-sn-glycerol 3-phosphocholine metabolites in serum, potentially affecting bone-related diseases and HIF-1 and glycolysis/gluconeogenesis metabolic pathways. Evidence from the included studies also implicated the gut and brain links in fluoride-induced neurotoxicity. Zhou et al. (2023) investigated fluoride exposure in Sprague-Dawley rats, finding changes in gut microbiota and energy metabolism pathways. Another experiment explored the effects of arsenic and/or fluoride exposure in Sprague Dawley rats, linking disruptions in amino acids and lipid metabolites to gut microbiota changes and learning and memory impairment (Zhang et al., 2023b). Dose- and generation-dependent alterations in synaptic vulnerability and neurotransmitter dysfunction were observed in C57BL/6 J mice exposed to fluoride, with significant changes in choline recycling and arachidonic acid metabolism pathways between parents and offspring (Zhu et al., 2024). Beyond systemic effects, fluoride was found to target specific organs, with toxicity exacerbated by co-exposure to other contaminants. Co-exposure to arsenic and fluoride in Sprague Dawley rats was found associated with disruptions in kidney function and autophagy, along with alterations in metabolites like glutamine, alpha-ketoglutarate, and phosphatidylethanolamine (Tian et al., 2023). Additionally, a study showed that dietary fluoride excess in Ross 308 broilers induced oxidative stress, lipid peroxidation, and iron accumulation, promoting ferroptosis in the liver (Wang et al., 2023a). In summary, these studies, encompassing various animal models and experimental designs, found alterations in metabolites and pathways related to kidney function, autophagy, amino acid metabolism, choline metabolism, glycerophospholipid metabolism, oxidative stress, lipid peroxidation, iron accumulation, gut microbiota, energy metabolism, synaptic vulnerability, and neurotransmitter dysfunction. Additionally, the studies highlight the relevance of factors such as co-exposure to other substances and dose-dependent effects.

Three in vitro experimental studies investigated alterations in cellular metabolism as a result of fluoride exposure on various cell types. The first study (Sakagami et al., 2014) focused on human oral squamous cell carcinoma (OSCC) cells, exposing them to sodium fluoride at a dose of 8 mM for varying durations. They observed changes in glycolysis and the tricarboxylic acid cycle, highlighting shifts in metabolites indicative of altered cellular metabolism. The second study (Wang et al., 2022) employed C57BL/6J mouse bone marrow stromal cells (BMSCs) and exposed them to sodium fluoride at doses of 1, 2, and 4 mM for 24 h, finding impaired lysosomal function, disruptions in glutathione and fatty acid metabolism, and altered metabolite levels, providing insights into mechanisms of fluoride-induced bone toxicity. Finally, the last study (Zhang et al., 2023a) used human umbilical vein endothelial cells (HUVECs) exposed to sodium fluoride at a dose of 75 µmol/L for 24 h. They found significant alterations in fatty acid and amino acid metabolism, suggesting potential pathways for fluoride-induced cardiovascular diseases.

### Synthesis of results

#### Metabolites

##### In-vitro studies

In human oral cancer cells, fluoride treatment led to an increase in several phosphate-containing metabolites, fructose 1,6-phosphate, 3-phosphoglycerate, dihydroxyacetone phosphate, 2-phosphoglycerate, and succinate, while lactate levels decreased significantly (Table 3). Metabolic alterations were observed in other cell types, with changes in amino acid metabolism involving L-glutamate, and lipid metabolism, evidenced by altered fatty acid and prostaglandin levels, indicating a broad impact on cellular metabolic functions (Sakagami et al., 2014; Wang et al., 2022; Zhang et al., 2023a).

##### Animal studies

In animal models, changes in the metabolic profiles were observed, including alterations in glutamine and alpha-ketoglutarate levels across various fluoride-exposed group, highlighting disruptions in metabolites such as proline, ornithine, betaine, and glutathione (Table 2). Other significant findings include variations in fatty acids and lipid metabolites, indicating differential generational and dose-dependent metabolic responses. This suggests an interplay of fluoride with metabolic regulation across different animal models and conditions (Ba et al., 2022; Tian et al., 2023; Wang et al., 2023a; Yue et al., 2020; Zhang et al., 2023b; Zhao et al., 2022; Zhou et al., 2023; Zhu et al., 2024).

##### Human studies

Studies on human serum and fecal samples revealed significant alterations in metabolites. A study of serum showed that metabolites such as inosine, α-linolenic acid, various forms of carnitine, and guanosine were generally downregulated in fluorosis compared to healthy controls (Table 1). Separately, a study on fecal samples found a significant decrease in tryptophan-derived metabolites, such as tryptamine and 5-hydroxyindoleacetic acid. These alterations point to a shift in metabolic processes (Usman et al., 2022; Wang et al., 2023b).

#### Metabolic pathways

##### In-vitro studies

The pathways most impacted by fluoride treatment included glycolysis, with inhibition of the enolase reaction reported in one study and general disturbance noted in another; the TCA cycle, showing initial activation followed by a decrease in specific metabolites; and energy metabolism, evidenced by altered ATP utilization (Table 3). Additionally, significant changes were noted in oxidative stress-related pathways, including those for glutathione metabolism, which was linked to enhanced oxidative stress and altered redox states in cells, and also associated with altered glutamic acid expression (Sakagami et al., 2014; Wang et al., 2022; Zhang et al., 2023a).

##### Animal studies

Pathway analyses in animal studies revealed alterations in several metabolic pathways (Table 2). For instance, some studies highlighted changes in the autophagy-animal pathway, phosphatidylethanolamine levels, and the pentose and glucuronate interconversion pathways, with significant shifts in α-ketoglutaric acid and UDP-glucose levels. Other studies identified disturbances in pathways to amino acid, lipid, and energy metabolism, such as urea cycle, choline metabolism, and the TCA cycle. Collectively, these findings reflect the metabolic disruption of animals to fluoride exposure (Ba et al., 2022; Tian et al., 2023; Wang et al., 2023a; Yue et al., 2020; Zhang et al., 2023b; Zhao et al., 2022; Zhou et al., 2023; Zhu et al., 2024).

##### Human studies

In humans, comprehensive pathway analyses indicated significant disturbances in pathways involved in purine degradation, fatty acid metabolism, and mitochondrial function (Table 1). These pathways exhibited decreased mitochondrial activity, changes in corticosteroid levels, and shifts in fatty acid signatures. These findings suggest metabolic disturbances associated with fluoride exposure. Other pathways, such as those related to protein digestion and absorption, and neuroactive ligand-receptor interactions, were also significantly affected, highlighting the broad potential impact of fluoride on human metabolic health (Usman et al., 2022; Wang et al., 2023b).

## Discussion

The findings of this scoping review indicate that chronic fluoride exposure may result in significant metabolic alterations, including dysregulation of key metabolites and pathways such as purine degradation, fatty acid metabolism, corticosteroids levels, protein digestion and absorption, and mitochondrial activity, implicating these alterations in the pathophysiology of fluorosis in humans. Animal studies support these findings. Fluoride exposure alters metabolites involved in amino acid, choline, and glycerophospholipid metabolism. It also disrupts multiple metabolic pathways, affecting organ function and metabolic processes.

### Human studies

In the present scoping review, fluoride exposure’s impact on metabolic pathways was found to be broad across the population groups studied and in-vitro cell models. A recurring finding was the alteration in lipid, amino acid and energy metabolism pathways. For instance, in human studies, there were findings of altered gut microbiota, carbohydrate metabolism dysregulation, and changes in fatty acid signatures. First, chronic fluoride exposure may significantly disrupt intestinal microflora and modify metabolite levels. This assertion is supported by evidence of changes in microbial diversity and structural damage to the intestinal barrier, which may directly affect gut health and metabolic integrity (Liu et al., 2019). Second, the mechanisms by which fluoride might influence carbohydrate metabolism dysregulation are complex and the evidence in humans is scarce, but evidence from microbiology studies points out at the disruption of key enzymatic pathways involved in carbohydrate metabolism in bacteria (Hamilton, 1977) and its influence on biofilm’s glucose uptake (Balzar Ekenbäck et al., 2001). Third, studies on the impact of fluoride on fatty acid signature are also limited in humans, with research from animal models suggesting that fluoride exposure can influence lipid synthesis pathways and cause oxidative stress, indirectly affecting fatty acid composition in some tissues (Suzuki et al., 2015). Overall, these alterations suggest the need for future studies on the systemic effect of fluoride on energy utilization and storage processes.

### Animal studies and In-vitro studies

In animal studies, similar findings were observed, with fluoride exposure leading to changes in serum metabolites related to amino acid and choline metabolism, disruptions in glycerophospholipid metabolism, and significant alterations in metabolic pathways linked to bone health and oxidative stress. The in-vitro studies further supported most of these findings, highlighting fluoride’s ability to disrupt fundamental cellular processes such as glycolysis, the tricarboxylic acid (TCA) cycle, and fatty acid metabolism. Earlier reports in the literature suggested that fluoride exposure may decrease the initial uptake and steady-state levels of amino acids in cells, indicating its potential to interfere with amino acid metabolism and signaling pathways essential for cellular function and metabolic integrity (Holland & Hongslo, 1979). More recent studies also support that fluoride exposure disrupts amino acid metabolism, as evidenced by alterations in the levels of neurotransmitter-related amino acids in the central nervous system, further delineating fluoride’s impact on metabolic and neurological functions (Zhu et al., 2024).

The specific pathways frequently cited across the studies in this scoping review include the purine degradation pathway, alterations in corticosteroid levels, protein digestion and absorption, glycerophospholipid metabolism, and pathways associated with oxidative stress and lipid peroxidation. Notably, the purine degradation pathway and lipid peroxidation mechanisms are central to cellular energy balance and redox homeostasis, respectively, with purine degradation linked to nitrogen recycling and signaling, while lipid peroxidation impacts cellular membrane integrity and signaling through the formation of bioactive molecules (Catalá, 2009; Hasegawa et al., 2008). These observations suggest a complex interplay between fluoride and its potential disruption of metabolic pathways.

### Summary

Many of the metabolic processes reported to be affected by fluoride exposure are interconnected, creating a web of influences that collectively contribute to the observed alterations in cellular function and overall metabolic integrity. Fluoride exposure leads to several interrelated changes in metabolism, including alterations in lipid metabolism, disruptions in amino acid and energy metabolism, and dysregulation of carbohydrate metabolism. It also impacts gut microbiota, influences the fatty acid signature, and causes changes in serum metabolites. Additionally, fluoride affects cellular processes and specific metabolic pathways. Understanding these interconnected responses is essential for elucidating the mechanisms behind fluoride’s effects.

Chronic fluoride exposure disrupts gut microbiota. This disruption may alter metabolic pathways that influence nutrient absorption and metabolism. This disruption in lipid metabolism can alter the composition of fatty acids in tissues, while changes in amino acid metabolism can affect neurotransmitter-related amino acids, ultimately impacting essential cellular signaling pathways. Furthermore, disturbances in energy metabolism can lead to dysregulation of carbohydrate metabolism, affecting how energy is used and stored in the body. The alteration of fatty acid signatures may be linked to oxidative stress caused by fluoride exposure, with lipid peroxidation potentially compromising cellular membrane integrity and signaling processes.

Changes in serum metabolites, including those related to amino acid and choline metabolism, indicate systemic effects on metabolic pathways. For example, disruptions in glycerophospholipid metabolism and alterations in pathways linked to bone health and oxidative stress are interconnected. Fluoride disrupts fundamental cellular processes, including glycolysis, the TCA cycle, and fatty acid metabolism, which can lead to broader effects on various metabolic pathways. Specific pathways that have been reported to be affected include the purine degradation pathway and mechanisms of lipid peroxidation. These pathways are crucial for maintaining cellular energy balance and redox homeostasis. Furthermore, the complex interactions with redox homeostasis suggest that metabolic changes induced by fluoride may have wider implications for cellular function and balance.

### Limitations

This scoping review has several limitations that should be considered. Firstly, the extrapolation of findings from in-vitro and animal studies to human contexts is challenging due to differences in doses, exposure conditions, and the biological variability in the response. Often, these studies employ concentrations that do not reflect typical human environmental or therapeutic exposures, which could lead to conclusions that are not directly applicable to human health, requiring cautious interpretation. Additionally, limiting our studies to those published in English, or with translations available in English, may exclude relevant research conducted in other languages. While the review included a broad range of bibliographic databases, the absence of databases specialized in certain geographical regions could further limit the comprehensiveness of our results. Therefore, the generalizability of our findings is likely restricted to the study populations from the information sources included and available for this review. Lastly, our results are current only up to February 2024.

## Conclusion

The evidence from the studies included in the present review suggests that fluoride exposure may significantly disrupt several key metabolic pathways mainly related to lipid, amino acid and energy metabolism, with potential implications for metabolic processes. Despite identifying 13 primary studies published between 2014 and 2024, our review highlights a notable lack of evidence in human studies, particularly regarding the mechanisms through which fluoride influences metabolic alterations. The higher availability of animal models and in-vitro studies provide valuable information for the design and conduct of studies in humans exposed to different levels of fluoride in the environment. Comparing metabolic alterations at different fluoride exposure levels in human populations would further our understanding of the systemic effects of fluoride on human health.

## Supplementary Information

Below is the link to the electronic supplementary material.

Supplementary Material 1

## Data Availability

No datasets were generated or analysed during the current study.

## Declarations

### Research involving human and animal participants

This article does not contain any studies with human and/or animal participants performed by any of the authors.
