# Supplementary material for: Fluoride exposure and metabolic alterations: a scoping review of metabolomic studies
Source: Metabolomics. 2025 Oct 10;21(5):147. doi: 10.1007/s11306-025-02353-w (PMC12513868; doi:10.1007/s11306-025-02353-w)
Supplement: Supplementary file 1 — Supplementary Material 1 [file 11306_2025_2353_MOESM1_ESM.docx]

**Date of last search:** February 6, 2024

**General search strategy**

(fluoride* OR fluorine*) AND (metabolomic* OR metabonomic* OR metabolome* OR metabolic profile*)

| **Database** | **Search strategy** | **Number** |
| --- | --- | --- |
| PubMed | ((fluoride*[Title/Abstract] OR fluorine*[Title/Abstract]) AND (metabolomic*[Title/Abstract] OR metabonomic*[Title/Abstract] OR metabolome*[Title/Abstract] OR metabolic profile*[Title/Abstract])) | 83 |
| Embase | ('fluoride':ab,ti OR 'fluorine':ab,ti) AND ('metabolomic':ab,ti OR 'metabonomic':ab,ti OR 'metabolome':ab,ti OR 'metabolic profile':ab,ti) | 59 |
| Scopus | (TITLE-ABS("fluoride*") OR TITLE-ABS("fluorine*")) AND (TITLE-ABS("metabolomic*") OR TITLE-ABS("metabonomic*") OR TITLE-ABS("metabolome*") OR TITLE-ABS ("metabolic profile*")) | 89 |
| Web of Science | (TS=("fluoride*") OR TS=("fluorine*")) AND (TS=("metabolomic*") OR TS=("metabonomic*") OR TS=("metabolome*") OR TS= ("metabolic profile*")) | 104 |
| Open Grey | fluoride OR fluorine AND metabolomic OR metabonomic OR metabolome OR "metabolic profile" | 0 |
